# Psychometric Testing of the Modified Dental Anxiety Scale among Iranian Adolescents during COVID-19 Pandemic

**DOI:** 10.3390/ejihpe11040092

**Published:** 2021-10-14

**Authors:** Chung-Ying Lin, Maryam Tofangchiha, Janneke F. M. Scheerman, Santosh Kumar Tadakamadla, Vijay Kumar Chattu, Amir H. Pakpour

**Affiliations:** 1 Institute of Allied Health Sciences, National Cheng Kung University Hospital, College of Medicine, National Cheng Kung University, Tainan 701401, Taiwan; cylin36933@gs.ncku.edu.tw; 2 Social Determinants of Health Research Center, Research Institute for Prevention of Non-Communicable Diseases, Qazvin University of Medical Sciences, Qazvin 3419759811, Iran; Mt_tofangchiha@yahoo.com; 3 Department Oral Hygiene, Inholland University of Applied Sciences, Cluster Health, Sport and Welfare, 1081LA Amsterdam, The Netherlands; janneke.scheerman@inholland.nl; 4 School of Medicine and Dentistry & Menzies Health Institute Queensland, Griffith University, Gold Coast 4222, Australia; santoshkumar.tadakamadla@alumni.griffithuni.edu.au; 5 Division of Occupational Medicine, Department of Medicine, Temerty Faculty of Medicine, University of Toronto, Toronto, ON M5C 2CS, Canada; 6 Department of Public Health, Saveetha Medical College, Saveetha Institute of Medical and Technical Sciences, Saveetha University, Chennai 600077, India; 7 Department of Nursing, School of Health and Welfare, Jönköping University, Gjuterigatan 5, 553 18 Jönköping, Sweden

**Keywords:** adolescent, dental anxiety, dental fear, Iran, psychometric properties

## Abstract

(1) Background: The present study aimed to examine the psychometric properties of the Persian adaptation of the Modified Dental Anxiety Scale (MDAS) in Iranian adolescents. (2) Methods: Adolescents with a mean age of 15.10 (n = 3197; 47.1% males) were recruited from Qazvin city of Iran using a stratified cluster random sampling technique. All children completed the five-item Persian MDAS and information related to background characteristics. Psychometric testing was conducted using classical test theory (CTT) and Rasch models. For CTT, an item-total correlation of >0.4 was considered satisfactory while for Rasch analysis, infit and outfit mean squares (Mnsq) ranging from 0.5–1.5 were considered satisfactory. Confirmatory Factor Analysis (CFA) was conducted to confirm the unidimensional structure of MDAS using various fit indices. Differential item functioning (DIF) was evaluated based on gender and time since last dental visit. Moreover, latent class analysis (LCA) was used to classify the participants into different levels of dental fear based on their pattern of responses. Both item level reliability using Cronbachs alpha (α) and test-reliability using intraclass correlation coefficients were evaluated. (3) Results: Item-total correlations ranged from 0.69–0.78, infit MnSq ranged from 0.80 to 1.11 and the range of outfit MnSq was 0.84–1.10. The data confirmed a one-factor structure of MDAS with satisfactory fit indices. DIF analysis indicated that the scale was interpreted similarly across the genders and time since dental visit groups. LCA analysis identified three levels, low, moderate and high levels of dental anxiety. The groups with moderate and high levels of dental anxiety had more females (44.6% and 36.7%) than the group with low level of dental anxiety (18.8%; *p* < 0.001). α of the total scale was 0.89 and item test-retest reliability ranged from 0.72–0.86. (4) Conclusions: The Persian MDAS was unidimensional with satisfactory psychometric properties evaluated using both CTT and Rasch analysis among Iranian adolescents. The scale was stable across the genders and individuals with different dental visiting patterns. The Persian MDAS also demonstrated excellent reliability.

## 1. Introduction

The novel coronavirus disease 2019 (COVID-19) hugely impacts individuals’ psychological health and overall wellbeing worldwide [1,2,3,4,5,6]. Social distancing and other public health measures prohibiting social interactions (e.g., city lockdown and school closure) are being implemented by several governments to mitigate the spread of the infection [7,8,9]. Under such circumstances, accessing health care and seeking treatment could be associated with significant psychological distress due to several reasons. First, due to the pain and discomfort associated with the health problems, second, due to the limited availability of health care services and lastly, the fear of risk of COVID-19 infection. More specifically, oral diseases and their treatment could cause significant stress and anxiety during the COVID-19 pandemic. This is because of the high risk of transmission of COVID-19 in dental practices due to the nature of dental treatment procedures and closer proximity of patient with the provider [10]. For these reasons, there is a greater tendency among individuals to evade dental treatment. This is more so among individuals with dental anxiety who are already more likely to avoid care.

Despite the high risk of cross-infection, adequate protection protocols could help ensure no transmission happens in a dental practice, there is no epidemiological evidence to support that high rates of cross-infection occurs in dental practices. Therefore, oral health care shouldn’t be evaded. It is important that oral health professionals and associated stakeholders adequately identify individuals with dental anxiety [11,12,13,14,15]. 

To date, one of the commonly used instruments assessing dental fear is the Modified Dental Anxiety Scale (MDAS), which was adapted and refined from the Corah’s Dental Anxiety Scale (CDAS) [16]. Moreover, the MDAS has been translated into 22 different languages [13]. Additionally, most psychometric evaluation studies on MDAS show that the MDAS has promising psychometric properties across different populations, including children [13,17,18,19,20]. For example, its unidimensionality has been supported in the Arabic version among an adult population [17]; its test–retest reliability and internal consistency are satisfactory in the Italian version among a pediatric population [20]; its criterion validity in the Japanese version is confirmed among an outpatient population [13]. 

The MDAS has been translated into Persian to assess the prevalence of dental anxiety [21]. Although some basic information on the psychometric properties (e.g., internal consistency) has been reported for the Persian MDAS [21], to the best of our knowledge, no study conducted an advanced analysis to evaluate the psychometric properties of MDAS in Persian or other languages. Table 1 presents the existing psychometric evidence on MDAS from its adaptation in different languages.

The psychometric properties of the MDAS tested in other language versions [13,17,18,19,20] cannot guarantee its robustness in assessing dental anxiety for Iranian adolescents. It is possible that the fear or distress associated with COVID-19 might impact the perception of dental anxiety. Hence, the information regarding the psychometric properties of the Persian MDAS during the COVID-19 pandemic is important for oral health professionals to know whether the outcomes assessed using MDAS are trustable. Given the context and the literature gap, the present study aimed to examine the psychometric properties of the Persian MDAS, using two types of psychometric theories [classical test theory (CTT) and modern test theory using Rasch models], among Iranian adolescents during the COVID-19 pandemic period.

## 2. Materials and Methods

### 2.1. Participants

The study participants were Iranian adolescents studying at high schools in Qazvin city (Iran). The inclusion criteria for the participants included (i) being an Iranian, (ii) studying in a high school in Qazvin, and (iii) being aged between 13 and 18 years. There were no other criteria for the eligibility and 3197 were involved (mean ± SD age = 15.10 ± 1.62 years; 47.1% males; mean ± SD fathers’ educational year = 9.79 ± 5.11; mean ± SD mothers’ educational year = 7.74 ± 5.01). The study was approved by both the Ethics Committee of Qazvin University of medical sciences (IR.QUMS.REC.1400.158) and the Organization for Education at Qazvin. All adolescents and their parents (one of their parents) had to read study aims and description and agree to participate before completing the study questionnaire. 

### 2.2. Procedures

**Figure 1:**
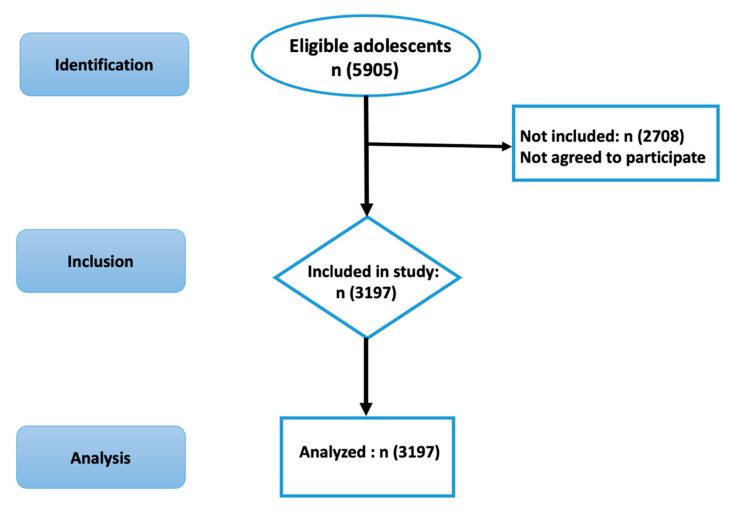
STROBE flow diagram showing the participant recruitment.

A stratified cluster random sampling procedure was used to recruit study sample. Qazvin has 3 education zones, and the sampling procedure was stratified based on these educational zones. A list of all high schools in Qazvin was used (provided by Organization for Education at Qazvin) to randomly select six high schools from each zone. Subsequently, all adolescents from each of the eighteen high schools were invited through Students Educational Network (SHAD) to participate in the study. Data were collected online using the survey administered on SHAD. SHAD is an online network for education and research used by all schools in Iran. All the participating adolescents were invited to complete the study measure after two weeks again. Around 72% of the adolescents (n = 2307) completed the study measure for the second time. The research process is presented in the flowchart of Figure 1.

### 2.3. Measures

The online survey comprised questions related to socio-demographic characteristics and oral hygiene practices along with the Persian version of the modified Dental Anxiety Scale (MDAS). The MDAS uses a five-point Likert scale on five items to assess the level of dental anxiety. A higher MDAS score indicates that the individual has higher levels of dental anxiety and the MDAS score ranges between 5 and 25 [19]. The reliability of the MDAS was found to be good: Cronbach’s α = 0.89; test–retest reliability = 0.82 [19]. The MDAS has been translated into Persian for use in Iranian population and a previous study showed that the internal consistency of Persian MDAS is satisfactory (Cronbach’s α = 0.80) [21]. 

The participants’ socio-demographic information such as age, gender, number of family members, number of years of education accomplished by father and mother were also collected. Oral hygiene practices included, time since last visit to a dentist, frequency of toothbrushing, and frequency of using a dental floss. 

### 2.4. Data Analysis

The demographic characteristics and oral hygiene practices of the participants were analyzed using descriptive statistics, including mean (SD) and frequency (percentage). Then, the psychometric properties of the MDAS were analyzed using both Classical Test Theory (CTT) and Rasch models. For CTT, internal consistency, corrected item-total correlations, test–retest reliability, exploratory factor analysis (EFA), confirmatory factor analysis (CFA), multigroup CFA, and known-group validity were tested. More specifically, internal consistency was checked via Cronbach’s α and McDonald’s ω, where >0.7 indicates satisfactory [22]. The corrected item-total correlation was also calculated, where a value of >0.4 indicates satisfactory [22]. Test–retest reality was examined via intraclass correlation coefficients, where a value of >0.5 indicates moderate and >0.75 indicates good [23]. 

CFA was also carried out to examine whether the MDAS fits a proposed one-factor structure. A diagonally weighted least squares (DWLS) estimator was applied in the CFA to fit the data and hypothesized structure. Several fit indices were used to examine the data-model fit in the CFA: comparative fit index (CFI) > 0.9, Tucker–Lewis index (TLI) > 0.9, root mean square error of approximation (RMSEA) < 0.08, and standardized root mean square residual (SRMR) < 0.08 [24,25]. Alongside the CFA, composite reliability (acceptable value > 0.6) and average variance extracted (acceptable value > 0.5) were calculated using the CFA factor loadings [26]. 

When the factor structure of the MDAS was confirmed, multigroup CFA with four nested models (configural model, metric invariance model, scalar invariance model, and strict invariance model) was conducted to examine the measurement invariance of the MDAS across gender and dental visit (i.e., those who have visited a dentist in the past six months vs. those who did not visit a dentist in the past six months). The configural model had no constraints on any item loadings and item thresholds for each group while metric invariance model was based on configural model to constrain the item loadings across groups; scalar invariance model was based on metric invariance model to constrain the item intercepts across groups; and strict invariance model was based on scalar invariance model to constrain the residuals across groups [27]. With the use of suggestions from [28], the measurement invariance was supported if the following indices satisfied: a nonsignificant χ^2^ test, ∆CFI > −0.01, ∆SRMR < 0.03 (for factor loading) or < 0.01 (for item intercept), and ∆RMSEA < 0.015.

In the Rasch models, two types of mean squares (MnSq)—inlier-sensitive (infit) and outlier-sensitive (outfit) MnSq—were used to assess MDAS item score. The acceptable value for both types of MnSq should be between 0.5 and 1.5 [26]. Moreover, differential item functioning (DIF) with a DIF contrast below 0.5 indicates that different gender groups and groups with different dental visit frequencies (i.e., visited a dentist in the past six months vs did not visit a dentist in the past six months) interpreted the MDAS items similarly [25]. Rasch models also reported separation reliability (including item separation and person separation) for the MDAS, and a value above 0.7 indicates satisfactory separation reliability [29]. Additionally, separation index (including item separation and person separation) was calculated from Rasch models to assess the MDAS score, and a value above 2 indicates satisfactory separation index [29].

The known group validity of the MDAS was examined using latent class analysis (LCA) because the LCA uses a person-centered approach to classify the participants into different levels of dental anxiety based on their pattern of responses. More specifically, the LCA used the Lo–Mendell–Rubin’s likelihood ratio test (LMR), entropy with Bootstrap Likelihood Ratio Test, the Bayesian information criterion (BIC), the Akaike information criterion (AIC), and the sample-size adjusted Bayesian information criterion (SSABIC), was used to determine the most appropriate number of levels among the participants. Among the different statistical parameters, AIC, BIC, and SSABIC are expected to be low, and the entropy is expected to be high. After determining the number of dental anxiety levels, analysis of variance (ANOVA) or χ^2^ tests were used to investigate whether different levels of dental anxiety have different features. The research process is presented in the flowchart below (Figure 1).

## 3. Results

Among 3197 participants (mean age was 15.1 years and 47.1% were males), less than a third (31.3%) have visited a dentist in the past year, and nearly one-fifth (18.0%) have never visited a dentist. Regarding their teeth cleaning behaviors, less than a fifth (19.1%) reported of toothbrushing twice a day, and nearly a tenth (8.4%) of the participants never used a toothbrush. Less than a sixth (15.9%) of children used dental floss while nearly half (49.5%) have never used dental floss (Table 2).

All the MDAS item scores demonstrated a normal distribution (range of skewness = −0.85 to −0.16; range of kurtosis = −1.09 to −0.18). Moreover, other item properties were satisfactory for all the MDAS items. In the properties derived from CTT, factor loading ranged between 0.87 to 0.98; corrected item to total correlation ranged from 0.69 to 0.78; and test–retest reliability ranged from 0.72 to 0.86. In the properties derived from Rasch analysis, infit MnSq ranged from 0.80 to 1.11; the range of outfit MnSq was 0.84 to 1.10; and DIF values across gender were −0.09 to 0.09 and −0.10 to 0.13 for dentist visit (Table 3). Apart from item properties, the scale properties of the MDAS were all satisfactory: composite reliability = 0.975; average variance extracted = 0.887; Cronbach’s α = 0.887; McDonald’s ω = 0.851; separation reliability from Rasch = 1.00 (for item separation) and 0.83 (for person separation); separation index from Rasch = 19.87 (for item separation) and 2.23 (for person separation). The results of the EFA showed that the MDAS has a unidimensional structure explaining a variance of 62%. All factor loadings were higher than 0.70 (ranging from 0.72 to 0.87). Moreover, the data confirmed a one-factor structure of MDAS with satisfactory fit indices: CFI = 0.993; TLI = 0.987; RMSEA = 0.069; SRMR = 0.047 (Table 4).

The measurement invariance of the MDAS was additionally confirmed by the multigroup CFA. More specifically, no significant differences were found between the nested models of configural model, metric invariance model, scalar invariance model, and strict invariance model across gender (*p*-values = 0.57 to 0.97; ΔCFI = 0.000; ΔRMSEA = −0.011 to −0.007; ΔSRMR = −0.006 to 0.001) and time since last dental visit (*p*-values = 0.48 to 0.93; ΔCFI = 0.000; ΔRMSEA = −0.011 to −0.006; ΔSRMR = −0.006 to 0.001) (Table 5).

The LCA results suggested that the participants should be classified into three levels (AIC = 39,708.98, BIC = 40,085.32, SSABIC = 39,888.32, Entropy = 0.864, LMR test = 1476.572, *p* = 0.0337) (Table 5). The three levels could be identified as low, moderate and high levels of dental anxiety, participant features based on the dental anxiety level are demonstrated in Table 5. Most of the adolescents clustered in the moderate dental anxiety class, these participants had higher dental anxiety scores than those in the low dental anxiety class but lower than those in the high dental anxiety class. The classes did not differ in relation to the age of the adolescent, fathers’ and mothers’ educational level. The groups with moderate and high levels of dental anxiety had more females (44.6% and 36.7%) than did the group with a low level of dental anxiety (18.8%; *p* < 0.001). The groups with moderate and high levels of dental anxiety had fewer participants visiting a dentist in the past six months (90.5% and 90.3%) than did the group with a low level of dental anxiety (91.5%; *p* < 0.001) (Table 6).

## 4. Discussion

The present study showed that the Persian MDAS had promising psychometric properties among adolescent Iranians, even in the current COVID-19 pandemic situation. More specifically, psychometric testing based on CTT indicated that the Persian MDAS had normally distributed item scores, excellent internal consistency; satisfactory test–retest reliability; and unidimensionality across the genders and dental visiting practice groups. Psychometric testing based on Rasch models also supported the unidimensionality of the Persian MDAS and separation reliability was also excellent. In addition, the Persian MDAS was able to classify the participants into three levels of dental anxiety. 

The findings derived from the CTT analyses in the present study are in accordance with the existing literature. For example, the internal consistency of the MDAS was also found to be excellent for Japanese (α = 0.88) [13], Italian (α = 0.87) [20], British English (α = 0.89), and Nepali (α = 0.78) adaptations [18]. The unidimensionality of the MDAS observed in the study was also confirmed in the Arabic version of the MDAS [17], and the test–retest reliability of the MDAS was satisfactory in the Italian version (r = 0.80) [20] and the British English version (r = 0.82), similar to this study [19]. Moreover, the internal consistency findings in the present study echo the prior Iranian study that used the Persian MDAS (α = 0.80) [21]. Ultimately, the present study’s findings corroborate with other MDAS psychometric studies in different language versions [13,17,18,19,20]. Moreover, the present study demonstrated that the MDAS could be applied to the adolescent population, and this finding aligns with a prior study that used MDAS in the pediatric population [20]. 

In this study, we extended our psychometric evaluation to Rasch analysis. Despite both CTT and Rasch analyses being used for psychometric evaluation, they have different statistical assumptions [26,29]. Therefore, it is important to provide psychometric information from both analyses to ensure that the instrument is robust and rigorous [26,29]. To the best of the authors’ knowledge, no prior research has used Rasch models to investigate the psychometric features of the MDAS. Therefore, findings derived from Rasch models in the present study couldn’t be compared with the existing literature. Future studies should use Rasch models to evaluate the psychometric properties of MDAS in different languages. 

During the COVID-19 pandemic period, visiting a dentist may increase the risk of getting infected with COVID-19. Given that oral health is important for individuals’ overall health and quality of life [11,12], tackling dental anxiety or dental fear for adolescents during the COVID-19 pandemic period is more critical as the COVID-19 situation further exacerbates the anxiety in dentally anxious individuals. Therefore, it is imperative to identify adolescents with high dental anxiety to promote confidence and provide adequate support to these individuals, thereby preventing avoidance of care in the current COVID-19 situation [30]. For this purpose, use of validated instruments like ours are of utmost importance to accurately assess dental anxiety. Moreover, the Persian MDAS contains only five items, and its administration requires a very short time. This feature further allows wider use of Persian MDAS in clinical as well as epidemiological contexts. 

### Limitations of the Study 

There are some limitations in the present study. First, the present sample is only representative of Iranian adolescents in Qazvin province, however, the sample size was large. Future studies are thus needed to test the psychometric properties of the Persian MDAS for Iranian adolescents residing in other areas to increase the generalizability of the present study’s findings. Second, we did not compare the Persian MDAS to any gold standard dental fear instruments to examine the criterion validity. Although all the psychometric testing indicated that the Persian MDAS has good reliability and validity, there is still a need to evaluate the criterion validity of the Persian MDAS. Third, the psychometric findings were derived from the data collected during the COVID-19 pandemic. Therefore, level of dental anxiety could have been overestimated by the study participants due to the COVID-19 pandemic. Future studies are warranted to examine the psychometric properties of the Persian MDAS when the COVID-19 pandemic is under control. 

## 5. Conclusions

The Persian MDAS was found to be unidimensional and demonstrated satisfactory psychometric properties evaluated using both CTT and Rasch analysis among Iranian adolescents. The scale was stable across the genders and individuals with different dental visiting patterns. LCA identified three distinct classes identified as, low, moderate and high levels of dental anxiety. The Persian MDAS scale demonstrated excellent internal consistency and all items had excellent test-retest reliability.

## Figures and Tables

**Table 1:** Psychometric evidence of the Modified Dental Anxiety Scale (MDAS) in the recent literature.

Authors	Year	Objective	Merits	Demerits	1	2	3	4	5	6	7
Ogawa et al. [13]	2020	1. Examine the reliability and validity of the Japanese MDAS. 2. Compare the prevalence of dental anxiety between Japan and other countries	Using CTT to test the psychometric properties for the Japanese MDAS among dental outpatients	Having no evidence in corrected item-total correlation, test–retest reliability, measurement invariance, known-group validity, and Rasch analysis.	Y	N	N	Y	N	N	N
Bahammam & Hassan [17]	2014	Examine the reliability and validity of the Arabic MDAS.	Using CTT to test the psychometric properties for the Arabic MDAS among dental outpatients	Having no evidence in test–retest reliability, factor analysis, measurement invariance, known-group validity, and Rasch analysis.	Y	Y	N	N	N	N	N
Giri et al. [18]	2017	Examine the reliability and validity of the Nepali MDAS.	Using CTT to test the psychometric properties for the Nepali MDAS among dental outpatients	Having no evidence in measurement invariance, known-group validity, and Rasch analysis.	Y	Y	Y	Y	N	N	N
Humphris et al. [19]	2009	1. Confirm factor structure of the MDAS. 2. Establish an UK norm of dental fear	Using CTT to test the psychometric properties for the British MDAS among general population	Having no evidence in corrected item-total correlation, test–retest reliability, measurement invariance, known-group validity, and Rasch analysis.	Y	N	N	Y	N	N	N
Paglia et al. [20]	2017	1. Examine the reliability and validity of the Italian MDAS for children version. 2. Compare the difference between child-rated and parent-rated MDAS. 3. Quantify the prevalence of dental fear among children.	Using CTT to test the psychometric properties for the Italian MDAS among pediatric population with the use of parent-proxy reports	Having no evidence in factor analysis, measurement invariance, and Rasch analysis.	Y	Y	Y	N	N	Y	N
Saatchi et al. [21]	2015	Evaluate the dental anxiety and fear in dental patients	Having the psychometric evidence of internal consistency in Iranian dental patients	Having no evidence in corrected item-total correlation, test–retest reliability, factor analysis, measurement invariance, known-group validity, and Rasch analysis.	Y	N	N	N	N	N	N

CTT = classical test theory. 1. Internal consistency. 2. Corrected item-total correlation. 3. Test–retest reliability. 4. Factor analysis for construct validity. 5. Measurement invariance. 6. Known-group validity. 7. Rasch analysis.

**Table 2:** Background characteristics of study participants (N = 3197).

	n (%) or Mean ± SD
Age	15.10 ± 1.62
Gender (male)	1507 (47.1%)
Number of family members	
≤4	2292 (71.7%)
5–7	552 (17.3%)
>7	353 (11.0%)
Years of education completed by father	9.79 ± 5.11
Years of education completed by mother	7.74 ± 5.01
Time since last dental visit	
<6 months	396 (12.4%)
6 months to 1 year	604 (18.9%)
1 to 2 years	797 (30.6%)
>2 years	641 (20.1%)
Never	577 (18.0%)
Frequency of toothbrushing	
Never	267 (8.4%)
Less than once per month	199 (6.2%)
Less than once per week	170 (5.3%)
Once per week	509 (15.9%)
Once per day	1442 (64.2%)
Twice per day	610 (19.1%)
Frequency of using dental floss	
Never	1583 (49.5%)
Less than once per month	334 (10.4%)
Less than once per week	323 (10.1%)
Once per week	451 (14.1%)
Once per day	506 (15.9%)

**Table 3:** Psychometric properties of the Modified Dental Anxiety Scale (MDAS) at the item level.

Item #	Analyses from Classical Test Theory					Rasch Analyses					
	Factor Loading *^,†^	Item-Total Correlation	Test–Retest Reliability ^‡^	S	K	Infit MnSq	Outfit MnSq	Difficulty	Discrimination	DIF Contrast across Gender ^§^^,¶^	DIF Contrast across Dental Visit ^§^^,#^
MDAS1	0.870	0.717	0.721	−0.854	−0.176	1.01	1.00	−0.76	0.99	−0.10	−0.10
MDAS2	0.981	0.782	0.779	−0.788	−0.294	0.80	0.84	−0.52	1.20	−0.09	−0.08
MDAS3	0.919	0.692	0.812	−0.157	−1.090	1.06	1.08	0.49	0.92	0.09	0.13
MDAS4	0.962	0.705	0.863	−0.364	−0.880	1.11	1.10	0.28	0.89	0.02	0.00
MDAS5	0.973	0.737	0.794	−0.232	−0.937	0.91	0.93	0.51	1.05	0.00	0.02

* All factor loadings were significant at 0.001. ^†^ Based on the first-order confirmatory factor analysis (CFA). ^‡^ Using Intraclass Correlation Coefficient (ICC). ^§^ DIF contrast > 0.5 indicates substantial DIF. ^¶^ DIF contrast across gender = Difficulty for males/Difficulty for females. ^#^ DIF contrast across anxiety = Difficulty for adolescents who visited a dentist in the past six months/Difficulty for adolescents not visited a dentist in the past six months. MnSq = mean square error; DIF = differential item functioning; S = Skewness; K = Kurtosis.

**Table 4:** Psychometric properties of the Modified Dental Anxiety Scale (MDAS) at the scale level.

Psychometric Testing	MDAS
Composite Reliability	0.975
Average Variance Extracted	0.887
Internal consistency (Cronbach’s α)	0.887
Internal consistency (McDonald’s ω)	0.851
Item separation reliability from Rasch	1.00
Item separation index from Rasch	19.87
Person separation reliability from Rasch	0.83
Person separation index from Rasch	2.23

CFA model fit: χ^2^ (*df*) = 80.348 (5); comparative fit index = 0.993; Tucker–Lewis index = 0.987; root mean square error of approximation (90% CI) = 0.069 (0.056,0.082); and standardized root mean square residual = 0.047.

**Table 5:** Measurement invariance across gender and time since last dental visit.

	Configural Model	Metric	Scalar	Strict
**Gender (males vs. females)**				
Χ^2^(df)/(ΔΧ^2^ [Δdf])	80.531 (10)/--	82.074 (14)/1.543 (4)	85.011 (18)/2.937 (4)	85.924 (23)/0.913 (5)
*p*-value for ΔΧ^2^	--	**0.82**	**0.57**	**0.97**
CFI/(ΔCFI)	0.994/--	**0.994/0.000**	**0.994/0.000**	**0.994/0.000**
RMSEA/(ΔRMSEA)	0.066/--	**0.055/−0.011**	**0.048/−0.007**	**0.041/−0.007**
SRMR/(ΔSRMR)	0.047/--	**0.048/0.001**	**0.042/−0.006**	**0.042/0.000**
**Time since last dental visit (adolescents visited a dentist in the past six months vs. adolescents not visited a dentist in the past six months)**				
Χ^2^(df)/(ΔΧ^2^ [Δdf])	80.342 (10)/--	81.210 (14)/0.868 (4)	84.721 (18)/3.511 (4)	88.676 (23)/3.955 (5)
*p*-value	--	**0.93**	**0.48**	**0.56**
CFI/(ΔCFI)	0.994/--	**0.994/0.000**	**0.994/0.000**	**0.994/0.000**
RMSEA/(ΔRMSEA)	0.066/--	**0.055/−0.011**	**0.048/−0.007**	**0.042/−0.006**
SRMR/(ΔSRMR)	0.048/--	**0.048/0.000**	**0.042/−0.006**	**0.043/0.001**

CFI = comparative fit index; RMSEA = root mean square error of approximation; SRMR = standardized root mean square residual. the **bold value indicates invariance**; i.e., ΔCFI > −0.01; ΔRMSEA < 0.015; ΔSRMR < 0.03 (for factor loading) or < 0.01 (for item intercept).

**Table 6:** Latent class analysis to identify subgroups of participants.

	AIC	BIC	SSABIC	Entropy	LMR Test (*p*-Value)
Class 1	47,710.753	47,832.152	47,768.604	n/a	n/a
Class 2	41,152.266	41,401.135	41,270.860	0.940	6561.768 (<0.0001)
Class 3	**39,708.981**	**40,085.319**	**39,888.319**	**0.864**	**1476.572 (0.0337)**
Class 4	39,011.515	35,515.322	39,251.596	0.875	735.128 (0.6277)

AIC = Akaike’s information criterion. BIC = Bayesian information criterion. SSABIC = sample-size adjusted BIC. LMR test = Lo–Mendell–Rubin’s likelihood ratio test.

## Data Availability

The datasets used for this research on adolescents cannot be shared with the public as per the privacy policy and regulations of Qazvin University of Medical Sciences. The data presented in this study are available on request from the corresponding authors.
